# Critical Analysis of Cytoplasmic Progression of Inflammatory Signaling Suggests Potential Pharmacologic Targets for Wound Healing and Fibrotic Disorders

**DOI:** 10.3390/biomedicines12122723

**Published:** 2024-11-28

**Authors:** Michael L. Samulevich, Liam E. Carman, Brian J. Aneskievich

**Affiliations:** 1 Graduate Program in Pharmacology & Toxicology, University of Connecticut, Storrs, CT 06269-3092, USA; michael.samulevich@uconn.edu (M.L.S.); liam.carman@uconn.edu (L.E.C.); 2 Department of Pharmaceutical Sciences, School of Pharmacy, University of Connecticut, Storrs, CT 06269-3092, USA

**Keywords:** wound healing, cytokines, chronic wounds, inflammation, scleroderma, keratinocytes

## Abstract

Successful skin wound healing is dependent on an interplay between epidermal keratinocytes and dermal fibroblasts as they react to local extracellular factors (DAMPs, PAMPs, cytokines, etc.) surveyed from that environment by numerous membrane receptors (e.g., TLRs, cytokine receptors, etc.). In turn, those receptors are the start of a cytoplasmic signaling pathway where balance is key to effective healing and, as needed, cell and matrix regeneration. When directed through NF-κB, these signaling routes lead to transient responses to the benefit of initiating immune cell recruitment, cell replication, local chemokine and cytokine production, and matrix protein synthesis. The converse can also occur, where ongoing canonical NF-κB activation leads to chronic, hyper-responsive states. Here, we assess three key players, TAK1, TNFAIP3, and TNIP1, in cytoplasmic regulation of NF-κB activation, which, because of their distinctive and yet inter-related functions, either promote or limit that activation. Their balanced function is integral to successful wound healing, given their significant control over the expression of inflammation-, fibrosis-, and matrix remodeling-associated genes. Intriguingly, these three proteins have also been emphasized in dysregulated NF-κB signaling central to systemic sclerosis (SSc). Notably, diffuse SSc shares some tissue features similar to an excessive inflammatory/fibrotic wound response without eventual resolution. Taking a cue from certain instances of aberrant wound healing and SSc having some shared aspects, e.g., chronic inflammation and fibrosis, this review looks for the first time, to our knowledge, at what those pathologies might have in common regarding the cytoplasmic progression of NF-κB-mediated signaling. Additionally, while TAK1, TNFAIP3, and TNIP1 are often investigated and reported on individually, we propose them here as three proteins whose consequences of function are very highly interconnected at the signaling focus of NF-κB. We thus highlight the emerging promise for the eventual clinical benefit derived from an improved understanding of these integral signal progression modulators. Depending on the protein, its indirect or direct pharmacological regulation has been reported. Current findings support further intensive studies of these points in NF-κB regulation both for their basic function in healthy cells as well as with the goal of targeting them for translational benefit in multiple cutaneous wound healing situations, whether stemming from acute injury or a dysregulated inflammatory/fibrotic response.

## 1. Introduction

Wound healing is a vital and complex physiological response to injury employed to restore the integrity of damaged tissues across diverse organ systems [1]. Because of their accessible position for observation, experimental investigations, and clinical intervention, the wound healing process involving epithelial and underlying connective tissues is of great interest [2]. Epithelial tissue includes both the keratinized epithelium of the skin and the non-keratinized epithelium of moist regions, e.g., the oral cavity and esophagus. Connective tissue, in this respect, mostly involves fibrous compartments (fibroblasts producing collagen and other extracellular matrix (ECM) components), which, for the skin, are recognized in papillary and reticular layers. Skin keratinocytes and fibroblasts are acknowledged as individually important in situ for tissue and in cell culture model systems of normal and aberrant wound healing and fibrotic responses. Nevertheless, of increasingly recognized significance is their cell–cell cross-communication, resulting in their both secreting and responding to numerous cytokines, chemokines, and matrix proteins [3,4,5,6,7,8,9]. These numerous and varied signaling molecules then contribute to differing phases of the wound healing process. Given the likely relationship of aberrant wound healing to certain skin fibrotic disorders (as we will expand in Section 2.1), we will emphasize cutaneous tissue from here forward.

The canonical wound healing process comprises hemostatic, inflammatory, proliferative, and maturation/remodeling phases [2,10]. Hemostasis, or cessation of bleeding, is achieved via primary and secondary hemostatic processes. Primary hemostasis refers to the initial aggregation of platelets at the site of injury to form a barrier protecting the exposed endothelium in conjunction with brief arterial vasoconstriction [11]. Secondary hemostasis sees activation of both the longer intrinsic and shorter extrinsic clotting pathways, which converge with the proteolytic activation of clotting factor X into factor Xa [12]. The cascade of conversions subsequent to the activation of factor Xa is common to both the intrinsic and extrinsic pathways. These ultimately result in the activation of fibrinogen into fibrin as well as factors that bind the fibrin strands into a fibrin mesh; this mesh stabilizes the platelet plug formed during primary hemostasis.

**Figure 1:**
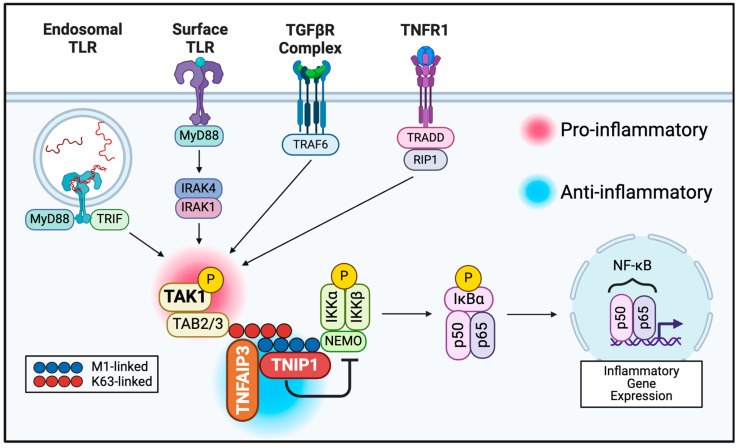
TAK1, TNFAIP3, and TNIP1 are focal point for regulating activation of NF-κB, leading to wound healing and fibrotic disorder gene expression (see text). In brief, function of TAK1 leads to progression of cytoplasmic signaling downstream of the indicated receptors, while TNFAIP3 and TNIP1 restrict continuation of the signal. Created in Biorender.com.

The inflammatory phase begins with the completion of the hemostatic stage, as thrombocytes involved in the process of hemostasis, along with resident skin cells such as keratinocytes, macrophages, dendritic cells, and mast cells are exposed to both damage- and pattern-associated molecular patterns (DAMPs and PAMPs, respectively) [13]. Pattern recognition receptors (PRRs) on the surface and endosomal membranes of these cells, e.g., Toll-like receptors (TLRs), recognize these DAMPs and PAMPs, triggering an intracellular inflammatory signaling cascade (Figure 1 and our review [14]). This leads to the release of cytokines and chemokines, which trigger local vasodilation and the chemotaxis of additional immune cells, e.g., neutrophils and monocytes, along with the continued inflammatory signaling of resident cells. Negative regulatory processes within cells prevent runaway inflammation, including the interruption of Transforming Growth Factor-β (TGF-β) Activated Kinase 1 (TAK1)’s interaction with polyubiquitin by the ubiquitin editing enzyme Tumor Necrosis Factor-α-Interacting Protein 3 (TNFAIP3, alias A20), whose function is facilitated by TNFAIP3-Interacting Protein 1 (TNIP1, alias ABIN1) (Figure 1). These proteins play a pivotal role in the regulating positive (TAK1) or negative (TNFAIP3, TNIP1) progression of NF-κB signaling. Nevertheless, they have scarcely been considered as therapeutic targets. Further, we suggest here that because of their inter-related but not redundant functions centered on NF-κB function, an integrated review of their role in that pathway, including the current, emerging, or potential opportunities to pharmacologically regulate them, could establish them as new targets for clinical modulation. As such, the cell physiological role and consequences of insufficiency and/or dysfunction of each of these proteins will be discussed in later sections.

The proliferative phase for cutaneous healing begins within a day of injury with the migration and proliferation of wound edge keratinocytes to cover the wound surface in a process called re-epithelialization. This expansion is driven by a lack of contact with the cells lost in the wound site, signaling cytoskeletal reorganization [15]. When the wound edge keratinocytes contact other keratinocytes, their migration ends, and they begin secreting ECM proteins contributing to the basement membrane [13]. It is also in this proliferative phase that fibroblasts migrate to the platelet plug and secrete proteases to remove it while also secreting components of the ECM to form granulation tissue. Finally, the maturation/remodeling phase sees the differentiation of fibroblasts in granulation tissue into myofibroblasts, which produce α-smooth muscle actin (α-SMA) to contract the wound [16].

Although brief, this overview of the classical four stages typical for effective cutaneous wound healing has nevertheless highlighted sufficient and progressive initiation and resolution of each phase [17]. As such, deficiencies or excesses at any stage can and do lead to chronic lesions of incomplete closure or hyper-responsiveness. The first of these two outcomes has been comprehensively reviewed [18,19,20]. Looking forward to the specifics presented below, we will survey excessive responses, especially those where NF-κB has been a focus of investigation. At the cellular and tissue level, this focus will lead us to unresolved, chronic inflammation, which so often accompanies transient dysregulated wound healing (see Section 2.1) and chronic fibrotic disorders (see Section 2.3), in particular for our consideration, systemic sclerosis (SSc, scleroderma).

## 2. Aberrant Wound Healing

### 2.1. Wound Healing Gone Wrong

Excessive inflammatory and fibrotic gene expression can underlie unsuccessful skin wound healing, contributing to chronic wounds. These endpoints may arise from diverse conditions of poor glucose regulation associated with diabetes and foot ulcers, surface compression leading to pressure ulcers on bony prominences, and chronic venous insufficiency predisposing to stasis ulcers on the lower extremities [17,21,22,23,24]. With regard to the inflammatory phase, there is something of a “golden mean” in terms of reaching regulatory homeostasis; too little or too much inflammatory signaling results in chronic inflammation, with runaway inflammatory signaling potentially leading to fibrosis. Indeed, Longaker and colleagues [25] recently remarked on wounds either “under-healing” (i.e., showing significant delays or complete failure to resolve) or “over-healing” (marked by scarring and/or fibrosis).

The latter two stages of wound healing, proliferation and remodeling, are also of particular interest in the investigation of chronic wounds as these lesions are typified by failure to progress to these stages [26]. Nevertheless, even successfully closed wounds regularly only reach ~80% restored tensile strength and are almost always marked by scarring [27,28] with parallel bundles of collagen rather than a multidirectional matrix of proteins. In some cases, these scars can be “hypertrophic”, resulting from an imbalance of ECM deposition and degradation in the remodeling phase [28,29]. These share some similarities in appearance to “keloids” but are distinct in terms of boundary and morphology, which are less defined for keloids and not limited to the site of initial injury [30].

The major cell type at play in driving fibrotic outcomes is myofibroblasts [31]. Myofibroblasts are phenotypically fibroblasts that have highly elevated levels of α-SMA chiefly, although other contractile proteins like myosin heavy chain isoforms can be involved [16,32]. These cells may be the products of differentiated fibroblasts but have also been shown to originate from epithelial and endothelial cells, fibrocytes, and smooth muscle cells [33,34]. Their major functions are producing collagens type I and II and providing contractile force; while doing so, they release cytokines such as TGF-β and EGF, amongst others [35,36]. When wound resolution progresses healthily, myofibroblasts undergo apoptosis, and as such, resistance to apoptosis by myofibroblasts is associated with fibrotic disease [37].

Though myofibroblasts are seen as the principal drivers of wound resolution and fibrosis, the independent and cooperative roles keratinocytes and fibroblasts play should not be overlooked [5,16,31]. Keratinocytes, the major constituent cell of the epidermis, serve as the first line of physical defense against pathogens but also have diverse roles in wound healing. These include innate immune functions through surveying their local environment via PRRs, e.g., TLRs [38,39,40] and secretion of cytokines, chemokines, and matrix metalloproteinases (MMPs), all of which are endpoints of keratinocyte inflammatory signaling [38,40,41,42]. Cytoplasmic signaling pathways to get to these endpoints thus offer potential entry points for pharmacologic regulation, especially to address “under-” and “over-healing” in chronic wounds or cutaneous fibrotic disorders such as scleroderma.

### 2.2. Systemic Sclerosis—Scleroderma

Broadly defined, there are two major categorizations of tissue hardening and fibrosis referred to as scleroderma. The first mostly affects cutaneous tissues, i.e., localized scleroderma. The second is excessive and hardened matrix proteins subtending numerous internal epithelial layers (e.g., skin, gastrointestinal tract, and lungs) but also blood vessel walls (i.e., systemic sclerosis [43]). Given cutaneous involvement in both groups, we inclusively use the term systemic sclerosis (SSc).

SSc is a poorly understood, chronic pathology marked by inflammation and immune system activation, dysfunction in microvascular cells and neighboring fibroblasts, and excess collagen deposition (fibrosis) in the dermis and internal organs [9,44,45,46,47,48]. Within this overall description, there is significant patient-to-patient diversity, important not only for definitive clinical classification but also for patient prognoses. Connective tissue fibrosis, particularly within the dermis, leading to skin hardening, is prototypical for the disease. The etiology, clinical pathology, and cell biology of SSc suggest a “perfect storm” of pre-disposing genetic factors, environmental stressors, and multi-level, multicell signaling interactions coming together to trigger inflammatory signals, microvascular damage, and fibrotic responses where wound healing resolution fails to occur [9,44,45,46,47,48,49]. Reminiscent of the “over-healing” suggested for excessive matrix deposition of terminating regular wound healing, SSc has also been considered a fibrotic stage that fails to resolve [4,50,51], possibly in part driven by ongoing TLR stimulation from otherwise unremarkable levels of DAMPs and PAMPs.

Advances have been made in understanding cell–cell interactions in producing and responding to inflammatory and fibrotic signals in SSc as key to tissue decline in multiple and diverse organs [45,50,52]. Nevertheless, there are significant treatment shortcomings, too often with limited initial or ongoing benefit to individuals living with SSc [53,54,55]. In brief, current treatment approaches for SSc slow the progression of this multifaceted disorder but do little to halt it [47,56,57]. These tactics include diverse blood pressure medications to address narrowed or constricted blood vessels associated with skin and lung complications, steroidal and non-steroidal anti-inflammatories, immune system suppressants, and medications for gastrointestinal tract symptoms [47,57,58]. Such drugs can have significant negative side effects [59,60,61]. Some, like cyclo-phosphamide, raise concerns of carcinogenesis, limiting their consideration and duration [57]. Pharmacologic and biologic intervention of pathway signaling, e.g., nintedanib, a kinase inhibitor for VEGF, FGF, and PDGF receptors, and tocilizumab, a humanized anti-IL-6 receptor monoclonal antibody, hold some promise for certain aspects of SSc. Nevertheless, each of these two treatments is again associated with adverse reactions, such as gastrointestinal side effects and serious infections, respectively [57,62,63].

### 2.3. Care Cost Impacts of Deficient Wound Healing and Fibrotic Disorders

In addition to the cell biology and medical science challenges in achieving appropriate wound resolution, there are the collateral difficulties of significant health care costs for acute wounds, especially chronic ulcerative lesions and inflammatory/fibrosing disorders such as SSc. While valuable for some comprehensive evaluation of care costs, there is limited availability of broad-based retrospective studies and/or they may be dated or difficult to use for estimation of current costs [64,65,66]. Spending from federal health insurance alone for wound care was estimated at USD 28 billion in a 2018 report and possibly up to three times this depending on diagnosis code criteria [67]. Costs are further exacerbated by infection, patient age, and comorbidities [65].

Comprehensive and directly comparable studies of costs for fibrosing disorders are limited [68,69]. Importantly, by the nature of the pathology, care costs can extend beyond the skin because of frequent internal organ dysfunction such as lung fibrosis, pulmonary arterial hypertension, and heart fibrosis. Significantly higher annual medical costs (reflecting “healthcare resource utilization”) are reported for people living with SSc than those not experiencing it [70,71,72]. In 2023, individual patient costs were reported to reach USD 30,000 [73]. The personal quality of life reductions and financial costs have spurred the identification and development of new therapeutics capitalizing on distinct targets built into cell signaling pathways inherent in regulating SSc inflammatory and fibrotic responses. Therapeutic success in the SSc arena may have benefits translatable to over-healing, scarring and keloids, and, in general, wound healing.

### 2.4. Consideration of Novel Therapeutics

There is an omnipresent call for novel drug development in targeting pathways [74,75,76,77], which would have therapeutic relevance to aspects of the excess inflammation and matrix deposition shared in both aberrant wound healing and SSc. Longaker and colleagues recently noted wound healing is “the largest medical market without an existing small molecule/drug treatment” [78]. These patient care realities highlight the need for the basic science investigation of cell signaling pathways relevant to effective wound healing that would lead to therapeutics to complement or synergize with current approaches. There has been some advancement in this regard. For instance, the potential for wound healing benefit via the regulation of proteins involved in local signaling was demonstrated by inhibiting Yes-associated protein with verteporfin, which in turn prevented Engrailed-1 expression [79]. This leads to a reduction in the usual fibrosis associated with scarring during wound healing, which is causative of reduced tissue flexibility and mechanical strength [80,81]. The possibility of reducing fibrosis brought about by verteporfin investigations highlights what opportunities may arise from more repurposing of approved drugs and natural products [82,83]. Verteporfin is mostly known for its photosensitizing effect, but it is increasingly being recognized for affecting cytoplasmic signaling pathways [84,85].

Beyond small molecule drugs that themselves might serve to facilitate any stages of wound healing is a prior and building interest [83] in the delivery of those compounds in formulations including but not limited to planar patches, moldable and injectable hydrogels, and nanoparticles that would provide for their local delivery in higher concentrations and rates of release from the drug-containing matrix. These delivery strategies might make possible the effective use of repurposed drugs not otherwise practical because of solubility or adverse events from systemic administration. Lastly, it is also important to note that conceptualizing the source of signaling initiators should not just be limited to soluble factors and cell or infecting bacteria debris. Wound physical tension or pressure, i.e., mechanotransduction, yields biochemical mediators central to the inflammation upstream of fibrosis [86].

Small molecule approaches are also being examined to advance SSc treatment options. Preclinical and trial results, including work with JAK inhibitors ([87,88] for example) and cannabinoid type 2 receptor agonists [89,90,91,92,93], highlight opportunities for SSc therapeutics development well outside current protocols, e.g., steroidal and non-steroidal anti-inflammatories and broad-based chemical and biologic immune system suppressants. For instance, lenabasum, an oral non-immuno-suppressive cannabinoid type 2 receptor (CB2) agonist, had phase 3 trial results recently reported [70]. Although with strong support from preclinical investigations demonstrating a reduction in inflammation and associated fibrosis markers [71] along with being well tolerated [72,94], the study does not show efficacy in diffuse cutaneous systemic sclerosis (dcSSc) [70]. Results may have been obscured by participants’ ongoing treatment regimens, including immuno-suppressive therapies. Separately, there are 2024 preclinical results [73] with CB2 agonist HU-308 diminishing skin and lung fibrosis in a bleomycin fibrosis mouse model.

### 2.5. Cytoplasmic Regulation of Post-Receptor Signaling May Provide New Opportunities

Given the as-of-yet unmet need for novel anti-inflammatory and antifibrotic therapeutics, additional intracellular pathways post the activation of membrane receptors are also being considered. A prime target is the NF-κB inflammatory pathway downstream of TLR, TGF-βR, and TNF-R, among others as it has been linked to wound healing inflammation and fibrosis as well as to SSc [95,96,97].

As outlined in Figure 1, the central hub of NF-κB signaling is the IκB kinase (IKK) complex (proteins in green), consisting of IKK-α and -β, the effector serine–threonine kinases, and their regulatory subunit IKK-γ, known more commonly as NF-κB essential modulator (NEMO) [98]. This complex is common to all pathways of upstream stimulators of NF-κB signaling, including, but not limited to, the TLR, TNFR, and TGFBR pathways (shown in Figure 1) [98,99]. Upon phosphorylation of the IKK-α and -β subunits, this complex can then phosphorylate downstream machinery such as inhibitor of κB (IκB) family proteins such as IκBα (shown in pink). Subsequently, these proteins are ubiquitinated by E3 ubiquitin ligase complex SCFβTrCP and degraded by the proteasome [100]. This then allows the nuclear translocation of NF-κB homo- and heterodimers (which are sequestered in the cytoplasm in resting cells), allowing them to act as transcription factors for a plethora of pro-inflammatory genes [101]. The NF-κB family consists of five different monomers: NFKB1 (p50/p105), NFKB2 (p52/p100), RelA (p65), c-Rel, and RelB [96]; the p50 and p52 subunits must also be cleaved via the proteasome, constitutively or upon stimulus, from their p105 and p100 precursors, respectively, in order to be active [102,103,104]. The p50/p65 dimer is shown in Figure 1 and is commonly cited as the most abundant, although this can vary based on cellular context [105].

Myriad genes regulated by NF-κB transcription factors downstream of surface receptor signaling experience increased expression during the wound healing process [101,106,107]. For example, CCR2 is a chemokine receptor with activity critical to early vascular sprouting. It has been demonstrated to both have increased expression in the tissue of patients during wound healing post burn injury and result in deficient wound healing with its knockout in murine studies [108,109,110]. The SERPINE1 gene has also been shown to be highly upregulated following suction-blister wound infliction [111]. PAI-1, the protein encoded by the SERPINE1 gene, is instrumental in re-epithelialization through its roles in both cell movement and tissue remodeling [112,113]. Unsurprisingly, the inflammatory cytokine TNF-α has been shown to be rapidly upregulated in wounded tissues and directly impacts wound healing through its acceleration of wound re-epithelialization and neovascularization [114,115]. Since runaway signaling in the inflammatory phase of wound healing has been linked to chronic and fibrotic wounds, there has been an investigation into the inhibition of TNF-α as a therapeutic avenue [116]. NF-κB activation is, thus, a centralization point of diverse extracellular signals leading to activation of inflammatory and fibrotic signals. Below, we survey TAK1, TNFAIP3, and TNIP1 as cytoplasmic proteins overseeing that activation with an eye toward the pharmacologic potential of this pathway (Figure 1). Although beyond the immediate scope of this review, we nevertheless stress the importance of a holistic appreciation of these three proteins in other signaling pathways, e.g., those mediated by MAPK or those leading to necroptosis or mitophagy (for review, see [117,118,119,120]).

## 3. Association of NF-κB Signal Repressing Proteins with SSc

### 3.1. TAK1

TGF-β-activated kinase 1 (TAK1, alias MAP3K7) was first identified in mice as a unique member of the mitogen-activated protein kinase (MAPK) family, which participates in the mediation of TGF-β superfamily signaling [121]. The human homolog of TAK1 was later shown to activate kinases downstream in multiple modes of inflammatory signaling, such as the IL-1β, NF-κB, and multiple MAPK pathways [122,123]. Comprehensively, TAK1 is now understood to be a central component in both cell death and inflammatory signaling in a multitude of cell types and disease states [117,124,125].

In signaling paradigms such as the IL-1β, TLR, TNF-R, and non-canonical TGF-β pathways, receptor activation leads to the recruitment of E3 ubiquitin ligases such as TRAF6, which form polyubiquitin chains [126,127]. The TAK1 binding protein family (TAB), namely members TAB2 and 3, bind the ubiquitin chains and recruit TAK1 to phosphorylate and activate downstream components, such as the IKK complex in NF-κB signaling and MAPKs, to progress signaling (Figure 1) [127,128,129]. While inflammatory signaling may carry a negative connotation, components of such signaling, including TAK1, play important roles in normal cell processes such as cell proliferation and survival, innate immunity in pathogen defense, and myocardial function [123,130,131]. This is demonstrated especially in wound healing, wherein TAK1 in dermal fibroblasts supports their proliferation and survival, the deposition of ECM, and ultimately the contraction of healing wounds [132].

The contribution to fibroblast function that TAK1 possesses is, of course, dependent on a delicate balance between sufficient and excessive signaling. Dysregulated wound healing, characterized by abnormalities in the newly formed fibronectin matrix (namely the presence of fibronectin EDA and unfolded type III fibronectin, recognized as DAMPs) yields excessive inflammatory signaling in fibroblasts through the TLR4/TAK1 axis [133]. The effects of fibronectin DAMPs varied by fibroblast origin, with dermal fibroblasts responding to FnEDA with the induction of the IL-1β and CCL5 genes [133], the latter protein having been implicated in the progression of scleroderma [3].

TAK1 mediates both TLR and TGF-β signaling (Figure 1) making it an enticing target for multiple modes of tissue fibrosis [125,134]. It comes as no surprise that experimental small molecule inhibitors of TAK1, such as HS-276 [134,135], have shown promise by preventing TGF-β-stimulated collagen synthesis and myofibroblast differentiation in control skin fibroblasts as well as mitigating the profibrotic phenotype in SSc fibroblasts and bleomycin mouse models (see Asano et al. [136] for further discussion of TAK1 pharmacologic inhibition). Interestingly, the inhibition of TAK1 with HS-276 concomitant with TGF-β exposure in fibroblasts resulted in the upregulation of some protective anti-inflammatory genes, such as *TNFAIP3* [134]. This suggests a synergistic effect of TAK1 inhibition, calming the progression of inflammatory signaling while also enhancing cells’ ability to dampen it. While other inhibitors of TAK1 have been developed, they have not shown the selectivity and bioavailability needed for clinical application [137]. Notably, HS-276 boasts an oral bioavailability of >95% and strong selectivity for TAK-1 over close homologs [134,138]; as such, it recently gained a U.S. FDA Orphan Drug Designation as EYD-001 [139].

TAK1 inhibition may also prove useful for reducing or preventing scar formation given its activity in dermal fibroblasts with the myofibroblast phenotype [140]. Salinomycin and related polyether ionophore antibiotics have been shown to reduce the activation of TAK1 as well as p38 in this signaling cascade [141]. Additionally, TGF-β-stimulated NIH 3T3 mouse fibroblasts showed reduced proliferation and collagen expression when treated with angiotensin-converting enzyme inhibitors (ACEIs) like lisinopril with the concomitant suppression of TAK1 phosphorylation [142]. siRNA knockdown of proteins CPEB1/4 acts similarly in suppressing TAK1 and SMAD signaling and reduces the expression of myofibroblast markers (α-SMA) that contribute to scarring as well as inflammatory markers like TNF-α and IL-6 [143]. Knockdown of both CPEB1 and 4 was accompanied by a significant reduction in phosphorylation of TAK1, p65, and IκBα, further supporting the involvement of the TAK1-NF-κB inflammatory axis in this effect. Interestingly, exosomes from scar fibroblasts have been shown to induce a profibrotic phenotype in healthy skin fibroblasts via the TAK1 and SMAD pathways, suggesting that small numbers of fibrotic cells can influence larger populations at the site of a wound [144].

In sum, TAK1 is an important central component of both TLR and TGF-β signaling, key in both healthy and fibrotic cell signaling. Its pharmacologic inhibition may provide synergistic anti-inflammatory effects in multifactorial events like wound healing and fibrotic diseases like SSc. Having considered TAK1 as the key kinase driving inflammation and fibrosis, it is important to now give attention to proteins central to the repression of inflammatory signaling as yet unexplored pharmacologic targets.

### 3.2. TNFAIP3

TNF α-induced protein 3 (TNFAIP3, alias A20) was initially discovered due to its expression in response to TNF stimulation of endothelial cells, leading to its description as an inhibitor of TNF-induced apoptosis [145]. Further investigation elucidated this protective function as a result of TNFAIP3 repressing NF-κB inflammatory signaling downstream of various receptor signaling pathways (e.g., TNF-Rs or TLRs) [118,146]. It was through this finding that TNFAIP3 was recognized as a ubiquitin editing enzyme with ligase and deubiquitinase functions [147]. However, the catalytic N-terminal deubiquitinase domain is largely dispensable for the TNFAIP3 reduction in NF-κB-mediated signaling [14,148]. The anti-inflammatory and, thus, cyto-protective abilities of TNFAIP3 seem to stem primarily from its non-catalytic zinc finger (ZnF) 7 and ZnF 4 polyubiquitin (pUb)-binding domains, suggesting that non-enzymatic protein–protein interactions (PPIs) are key. Importantly [14,148], its overall effect may be dependent on cell type, expression levels, and availability of TNFAIP3-partner proteins, e.g., TNIP1 (Figure 1). The latter apparently can act independently of TNFAIP3, but also with it, to bring about a limitation to NF-κB activation [149], leading to the notion that non-enzymatic TNIP1 serves as a scaffold to facilitate TNFAIP3’s pUb interaction [119].

TNFAIP3 is known to stunt IKK activation by the TAK1:TAB2/3 heterodimer through its recognition of M1-linked (linear) pUb chains via its ZnF7 domain [150]; this interaction (Figure 1) is thought to be strengthened via TNIP1 interaction, as TNIP1 also preferentially recognizes linear pUb chains via its UBAN domain [151,152]. TNFAIP3’s prevention of IKK complex phosphorylation prevents subsequent activation of the NF-κB complex for nuclear localization, thus preventing further NF-κB-mediated inflammatory signaling [101].

The genes whose transcription is upregulated by NF-κB signaling are cell type-dependent, with the pro-inflammatory signaling seen in keratinocytes contributing to the inflammatory phase of wound healing and fibroblasts seeing an increase in not only pro-inflammatory gene expression but also profibrotic gene expression [153,154]. Thus, TNFAIP3-mediated repression of NF-κB signaling is a crucial braking mechanism for multiple phases in overactive wound healing [155].

The need for the crucial negative regulatory capacity of TNFAIP3 in typical wound healing is echoed in SSc as a hyper-inflammatory and fibrotic disorder. Perhaps most telling are the significantly reduced levels of TNFAIP3 mRNA in the transcriptome of SSc skin biopsies [156]. Additionally, making it something of an innate trait rather than some response to the tissue environment, *TNFAIP3* expression is decreased in explanted SSc fibroblasts when controlled against healthy explanted fibroblasts [156]. Less immediately obvious but not necessarily less important are *TNFAIP3* links to SSc by various genome-wide association studies (GWASs) and other gene polymorphism reports [157,158,159,160,161,162]. In a specific example, the SNP rs58905141 of *TNFAIP3* was reported as linked with the expression of MMPs in fibroblasts challenged with silica particles, an environmental factor linked with the development of SSc [163].

Some pharmacological regulation of TNFAIP3 levels, although outside the arena of wound healing, has been reported due to its inflammation-limiting effect in diverse cells and tissues. This appears to be through increasing its expression level, rather than outright small molecule binding to its protein and facilitating its function. For instance, ikarugamycin and quercetin [164] and gibberellic acid [165] induce the expression of *TNFAIP3* in culture models employing bronchial epithelial cell lines. This suggests the compounds’ relevance to minimizing the throughput of incoming inflammatory signals associated with cystic fibrosis. Highlighting the need to look beyond typical dermal wound healing reports, Sun and colleagues recently reported [166] on increased *TNFAIP3* expression in prostate cancer cells as a consequence of their exposure to the pyrimidine diamine compound ZY-444, also known as an inhibitor of pyruvate carboxylase.

Reduction in NF-κB activation by increased *TNFAIP3* expression was also central to inflammatory and fibrotic signal reduction after the stimulation of the adiponectin receptor in dermal fibroblasts. This was achieved initially with the 244-amino acid adipokine hormone and later with a small molecule ligand that binds to and activates the receptor [156,167,168]. The latter is advantageous to translational development for aberrant wound healing or SSc treatments because it would be orally available without the encumbrances of the active agent being a relatively large protein. These and other TNFAIP3 findings above do strengthen its positioning as a wound healing/fibrosis target, especially in the context that enhancing its protein levels for its deubiquitinase activity and/or PPIs may bring about the desired effect. One such PPI is with its partner protein TNIP1, which cooperatively facilitates TNFAIP3’s innate repression of inflammatory signaling [169,170,171].

### 3.3. TNIP1

The TNIP1 acronym derives from TNF α-induced protein 3-Interacting Protein 1, with TNFAIP3 being a TNIP1-binding protein. TNIP1 protein functions with and, importantly, independently of TNFAIP3 to significantly limit the cytoplasmic progression of inflammatory and fibrotic signals downstream of TLR and TNF-R, as we [39,172] and others reported [151,173]. In brief, TNIP1 terminates post-membrane receptor signals (Figure 1) by assembling protein–protein interactions (PPIs) [119,172,174,175]. This inhibition arises from the required TNIP1 recognition of linear pUb on other proteins and its non-compulsory association with partner protein TNFAIP3 for the termination of membrane receptors’ signal cascades, otherwise leading to NF-κB, p38 MAPK, or JNK activation with subsequent inflammatory and fibrotic responses. Physiologically normal levels of TNIP1 protein significantly limit (Figure 1) the cytoplasmic activation of NF-κB downstream of TLR and TNF-R stimulation in diverse cell types [39,119,171,172,176,177,178,179].

Despite the great potential to quell the cytoplasmic progression of inflammatory signaling, there are very limited TNIP1-dedicated wound healing studies to date. We previously reported [172] that TNIP1-deficient keratinocytes are hyper-responsive to the model DAMP / PAMP poly (I:C), with enhanced expression of wound-associated markers (e.g., TGF-β and CCN2). Nevertheless, in a cell wound healing model, there was restricted re-epithelialization and reduced cell viability, potentially due to increased priming of the inflammasome, suggesting the need for TNIP1’s limiting of TLR-initiated signaling early in and throughout the repair process.

In contrast to the paucity of TNIP1 studies in wound healing per se, it has been repeatedly cited [180,181,182,183,184,185,186] (and see our reviews [14,119]) over the last 13 years as one of the highest-scoring SSc risk loci amongst non-HLA genes in multiple populations from numerous GWASs. Importantly, within these studies are *TNIP1* risk loci where, when examined, its protein in SSc tissue was significantly reduced. This was paralleled by its mRNA and protein being reduced in cells cultured from SSc donors to about half that compared to control cells [180]. Separate expression array studies [187] support reduced TNIP1 in affected skin compared to patient-uninvolved skin. The findings are consistent with SNPs that may lessen transcription factor binding at the gene promoter or mRNA intron processing efficiency and/or mRNA half-life, leading to decreased mature mRNA and protein [188].

Regarding the TNIP1 functionality of NF-κB inhibition, this predicts hyper-responsiveness to stimuli activating TLR and TNF-R. Indeed, Allanore and colleagues reported [180] reduced *TNIP1* expression in fibroblasts cultured from SSc donors, where the cells produced elevated levels of collagen in response to a TNFα challenge. Importantly, for what may be a runaway loss of TNIP1 in instances where there is a genetic disposition to already reduced levels, TNIP1 is degraded, as we [189], and later others [190], showed, as a consequence of inflammation. Notwithstanding the extensively demonstrated repression of cytoplasmic progression of inflammatory signaling and its involvement in fibrotic responses, it is uncertain to date if TNIP1 will be successfully clinically targeted to augment its expression or function. In sum, TAK1, TNFAIP3, and TNIP1 are all involved in NF-κB cytoplasmic signaling in such a way that repression of TAK1 or facilitation of TNFAIP3 and TNIP1 could repress inflammatory signals.

## 4. Concluding Observations

The importance of integrated, multidisciplinary approaches to advance wound healing research is evidenced by the current, only partially met, need to achieve optimal or complete extents of tissue repair/regeneration in a timely fashion. This is the case for both recovery from acute injury (physical or thermal trauma) as well as those incidents that arise from persistent conditions (e.g., vascular insufficiency, compromised mobility, or diabetes) that lead to chronic lesions (e.g., venous and/or arterial, decubitus, or diabetic foot ulcers) [18,19] or ongoing hyper-inflammatory and hyperfibrotic disorders (e.g., SSc). For wound healing, current approaches have led to better physical and chemical control of infections along with synthetic and biological barriers to protect and better manage tissue voids while supporting tissue regrowth [22,191,192]. Nevertheless, shortcomings remain in our understanding of macro events as well as intracellular signaling both during sufficient wound healing and especially instances of imbalanced healing and fibrotic disorders.

New areas of research are being informed by investigating intracellular signaling pathways responsible for the discrete phases of both normal and dysregulated wound healing. Prominent in this respect are proteins promoting or restricting NF-κB, given its extensive history of research in balancing transient and chronic inflammation as well as beneficial or excessive matrix deposition [193,194,195,196]. In both of these instances, there are emerging ideas that studies of acute and chronic wounds as well as disorders of inflammation and fibrosis, when considered to be aberrant wound healing, can cross-inform each other [4,50,51,197,198]. Recalling the earlier cited comments of Longaker and colleagues [78] on the need for small molecule therapeutics development in this area, we suggest that small molecule inhibitors of TAK1 being investigated for limiting fibrosis in SSc may result in fruitful studies on the regulation of excess matrix deposition in scarring and keloid formation. Complementing this approach, the augmented expression of *TNFAIP3* being investigated in multiple avenues (e.g., SSc, cystic fibrosis) could prove to be productive grounds for dedicated wound healing studies capitalizing on the repurposing of approved or pipeline therapeutics. Lastly, interventions to block NF-κB for the expected advantages of supporting later wound healing phases will likely need to be balanced against desirable NF-κB-mediated outcomes, e.g., migration and/or proliferation [195].

## 5. Prospectus

A new understanding of the dynamics of wound healing can continue to inform therapeutic options for those experiencing chronic inflammatory or fibrotic disease states. Very recent work using specific cell ablation techniques [199] helps to call for a reconsideration of physical events outside the cell at the tissular level of the skin. They detected unexpected dynamic aspects within the classical wound healing phase progression. Their results support envisioning a pre-wound solid-like homeostatic state. Upon wound healing, a transient fluid-like state is brought about by changes in the local signaling environment that influence inflammation, cell migration, and replication. Ultimately, in successful wound healing, there is a return to a solid-like homeostatic state. The challenge remains now to bridge these newly appreciated tissue physical repair dynamics with the specific signaling pathways responsible for them and how to pharmacologically intervene to address signaling defects leading to the under- or over-healing described above.

In addition to reimagining the physical state of cells as above, there are similarly innovative approaches being taken in other arenas to facilitate wound healing. While outside the pharmacologic scope of this review, naturally derived and engineered biomaterials are being investigated for their ultimate effect of limiting NF-κB expression or its activation [200,201]. Going to the source of proteins, gene editing is also being considered for diverse elements of wound healing [202] including those that drive inflammation. Of particular note is an inflammation-induced CRISPR-Cas9 system [203], where the initiation of inflammatory signaling was itself used as a means to negate the expression of MyD88, an upstream signal relay point in eventual NF-κB activation. Parallel to this, non-coding RNAs and their involvement in the regulation of cell membrane receptor-NF-κB signaling axes are also under investigation [204].

An often-overlooked factor involved in the signaling cascades necessary for proper wound healing is polyubiquitin (pUb). We looked intracellularly at three specific proteins, TAK1, TNFAIP3, and TNIP1, for new opportunities in addressing cytoplasmic pathway regulation central to wound healing and fibrotic disorders. Intriguingly, pUb chains, whether part of protein–protein recognition and interaction or for targeting of protein degradation cellular machinery, affect the function of these three proteins. Polyubiquitin reading (recognition), writing (addition), and erasing (removal) have been functionally summed as responsible for the “ubiquitin code” [205,206]. Other proteins responsible for these functions are likely themselves to be yet more intracellular targets for eventual pharmacologic regulation (direct or indirect depending on the reading/writing/erasing action of the involved protein) to better fulfill phases of wound healing. For instance, neuronally expressed developmentally downregulated 4 (Nedd4) is an E3 ligase (writer) central to corneal epithelial wound healing. Although not yet small molecule-targeted, its natural progression of increased expression during epithelial repair suggests its pharmacologically enhanced expression or activity could further its positive effects in that tissue. Additionally, erasers (deubiquitinating enzymes) are being examined in animal cutaneous wound healing models where, at least for ubiquitin C-terminal hydrolase L1 (UCHL1) [207], inhibition by a small molecule promoted wound healing. Finally, the dedicated writing versus erasing functions of individual proteins such as Nedd4 and UCHL1 participating in the ubiquitin code recall that these two functions as well as pUb recognition have been recognized for TNFAIP3 [14]. It may be that those individual roles are more prominent at distinct points of wound healing as far as guiding their small molecule enhancement or repression.

There is a continuously expanding knowledge base of the dynamics of wound healing, especially from key signaling modulators, such as the TAK1, TNFAIP3, and TNIP1 proteins emphasized in this review. Despite their distinct individual abilities, their functions converge on one of the most universal points of inflammatory signaling in successful wound healing, dysregulated recovery, and fibrotic disorders: cytoplasmic activation of NF-κB. In each instance, their simultaneous consideration as offered in this review is likely to inform future therapeutics development, as is emerging with small molecule regulation, directly or indirectly, for at least some of them. Nevertheless, multipronged and integrated investigations of intracellular events like NF-κB activation and gene targeting should be synergized with extracellular events such as the conceptualization of a quasi-fluidic wound tissue and biomaterial effects to the benefit of realizing better clinical control. Merging these individual investigative avenues could provide a more therapeutically effective “superhighway” beyond the current armamentarium for treating wound healing and fibrotic disorders.
